# Myocardial Work Indices Predict Hospitalization in Patients with Advanced Heart Failure

**DOI:** 10.3390/diagnostics14111196

**Published:** 2024-06-06

**Authors:** Giulia Elena Mandoli, Federico Landra, Benedetta Chiantini, Lorenzo Bonadiman, Maria Concetta Pastore, Marta Focardi, Flavio D’Ascenzi, Matteo Lisi, Enrico Emilio Diviggiano, Luca Martini, Sonia Bernazzali, Serafina Valente, Massimo Maccherini, Matteo Cameli, Michael Y. Henein

**Affiliations:** 1 Department of Medical Biotechnologies, Division of Cardiology, University of Siena, 53100 Siena, Italybenedetta.chianti@student.unisi.it (B.C.); lorenzo.bonadiman@student.unisi.it (L.B.); pastore2411@gmail.com (M.C.P.); focardim@unisi.it (M.F.); flavio.dascenzi@unisi.it (F.D.); e.diviggiano@student.unisi.it (E.E.D.); lucamartini54@gmail.com (L.M.); seravalente@gmail.com (S.V.); matteo.cameli@yahoo.com (M.C.); 2 Institute of Public Health and Clinical Medicine, Umeå University, 901 87 Umeå, Sweden; michael.henein@umu.se; 3 Department of Cardiovascular Disease-AUSL Romagna, Division of Cardiology, Ospedale S. Maria Delle Croci, 48121 Ravenna, Italy; matteo.lisi@hotmail.it; 4 Department of Cardiac Surgery, University of Siena, 53100 Siena, Italy; s.bernazzali@gmail.com (S.B.); maccherini2@unisi.it (M.M.)

**Keywords:** speckle tracking, myocardial work, end-stage heart failure, prognosis

## Abstract

Background: An increasing proportion of heart failure (HF) patients progress to the advanced stage (AdHF) with high event rates and limited treatment options. Echocardiography, particularly Speckle Tracking-derived myocardial work (MW), is useful for HF diagnosis and prognosis. We aimed to assess MW’s feasibility in the prognostic stratification of AdHF. Methods: We retrospectively screened patients with AdHF who accessed our hospital in 2018–2022. We excluded subjects with inadequate acoustic windows; unavailable brachial artery cuff pressure at the time of the echocardiography; atrial fibrillation; and mitral or aortic regurgitation. We measured standard parameters and left ventricular (LV) strain (LS) and MW. The population was followed up to determine the composite outcomes of all-cause mortality, left ventricular assist device implantation and heart transplantation (primary endpoint), as well as unplanned HF hospitalization (secondary endpoint). Results: We enrolled 138 patients, prevalently males (79.7%), with a median age of 58 years (IQR 50–62). AdHF etiology was predominantly non-ischemic (65.9%). Thirty-five patients developed a composite event during a median follow-up of 636 days (IQR 323–868). Diastolic function, pulmonary pressures, and LV GLS and LV MW indices were not associated with major events. Contrarily, for the secondary endpoint, the hazard ratio for each increase in global work index (GWI) by 50 mmHg% was 0.90 (*p* = 0.025) and for each increase in global constructive work (GCW) by 50 mmHg% was 0.90 (*p* = 0.022). Kaplan–Meier demonstrated better endpoint-free survival, with an LV GWI ≥ 369 mmHg%. Conclusions: GWI and GCW, with good feasibility, can help in the better characterization of patients with AdHF at higher risk of HF hospitalization and adverse events, identifying the need for closer follow-up or additional HF therapy.

## 1. Introduction

In developed countries, the overall incidence of heart failure (HF) is increasing, and its prevalence is around 1–2% in the adult population [1,2,3,4]. Proportionally, the prevalence of patients reaching the advanced stage of the disease is rising [1,5]. The prognostic stratification of patients with advanced heart failure (AdHF) is essential to establish optimum therapeutic management and a follow-up strategy. However, due to the non-linear course of this disease’s pathology, currently, predicting the prognosis of HF patients is still challenging [1,5,6]. Echocardiography is one of the most useful investigations for the assessment of patients with HF. Even when measurements are not consistent, it has an optimal capability to predict clinical outcomes [7,8]. The left ventricular ejection fraction (LVEF) is the most used index for assessing systolic function and helps in the classification of HF. The prognostic performance of LVEF in HF could be influenced by multiple factors such as preload and afterload [9]. Speckle Tracking Echocardiography (STE)-derived global longitudinal strain (GLS) has demonstrated greater sensitivity in detecting abnormalities of LV contractile function, even in preserved EF, with higher reproducibility but a similar dependence on afterload [10]. Myocardial work (MW) is a relatively new echocardiographic tool which allows the integration of LV afterload with GLS. MW provides a deeper insight into myocardial performance by estimating pressure–deformation curves in a non-invasive way [10,11,12,13,14]. MW-derived parameters have demonstrated feasibility and applicability in patients with HF in different clinical contexts, among which are the characterization of patients with preserved LV EF [15], a correlation with invasively measured filling pressures [16] and support for mechanical circulatory support system optimization [17]. To the best of our knowledge, MW’s utility in predicting prognoses in AdHF patients has never been assessed. To evaluate the prognostic value of LV MW indices in a population of patients with AdHF, we tested the different indices for mid-term prognostic stratification.

## 2. Materials and Methods

### 2.1. Study Population

Consecutive patients with AdHF referred to our tertiary referral center for work-up screenings for heart transplants (HTx) or mechanical circulatory support systems (left ventricular assist devices, LVAD) between January 2018 and December 2022 were retrospectively screened. The exclusion criteria were inadequate echocardiographic acoustic windows for MW analysis or incomplete echocardiographic examination; unavailable brachial artery cuff pressure measured at the time of the echocardiography; atrial fibrillation; more than mild mitral or aortic regurgitation; and absent informed consent. The study was performed in accordance with the Declaration of Helsinki and was approved by the local Ethics Committee.

### 2.2. Data Collection

In this observational, monocentric and retrospective cohort study, we collected clinical, demographic and laboratory data obtained from the institution’s electronic records. All echocardiographic examinations were performed by experienced operators using a GE Vivid E80/E95 (GE Medical Systems, Northen Ireland) equipped with an adult 1.5–4.3 MHz phased-array transducer, and with a continuously traced ECG, according to the American Society of Echocardiography/European Association of Cardiovascular Imaging recommendations [18,19]. For STE analysis, the endocardial borders and the myocardium layer of all segments from the apical views (four chambers, two chambers and apical long axis) had to be clearly visualized throughout the whole cardiac cycle. Image analysis was retrospectively performed offline using EchoPAC software v204 (GE Medical, Milwaukee, WI, USA). Brachial artery cuff pressure was measured 15 min after the end of the echocardiographic examination with the patient lying in a calm and comfortable position [19].

### 2.3. Myocardial Work Analysis

The STE-LV strain was semi-automatically performed by the software in the three apical views and was manually adjusted by the operator, to optimize the region of interest’s (ROI) width and positioning to obtain accurate endomyocardial tracking. For MW analysis, markers of the aortic and mitral valves’ opening and closure were set at the beginning and the end of each main phase of the cardiac cycle from the apical long axis view. A brachial blood pressure cuff was inserted to adapt the reference curve in terms of time and amplitude for LV pressure estimation, and the one detected at the time of echocardiography. The software output provided the following parameters for all patients: global work index (GWI), the total work performed by the heart between mitral valve closure and mitral valve opening; global constructive work (GCW), the work performed during shortening in systole in addition to the work performed during lengthening in isovolumetric relaxation; global wasted work (GWW), the work performed during lengthening in systole in addition to the work performed during shortening in isovolumetric relaxation; and global work efficiency (GWE), which is the constructive work divided by the sum of GCW and GWW [20,21,22]. MW analysis was performed using EchoPAC software v204 (GE Medical, Milwaukee, WI, USA) by a single experienced operator to reduce biases.

### 2.4. Outcomes

Clinical outcomes were gathered from the electronic records. The electronic records were used to identify the survival status at the latest available follow-up. 

The primary endpoint was a composite outcome of all-cause mortality, left ventricular assist device implantation and heart transplantation. The secondary endpoint was unplanned HF hospitalization.

### 2.5. Statistical Analysis

Continuous data are presented as median and interquartile range or as mean and standard deviation, as appropriate. Categorical data are shown as absolute and relative frequencies. The *t*-test or the Mann–Whitney U test was used for the comparison between continuous variables, as appropriate. The chi-square test was used for comparing categorical variables. A Receiver Operating Characteristic (ROC) analysis was performed to identify cut-offs for predicting the outcome using the Youden index. Univariate and multivariate Cox proportional hazard regression analyses were applied to assess predictors of outcomes. Kaplan–Meier analysis was used to estimate event-free survival. A *p*-value < 0.05 was considered statistically significant. All analyses were performed using SPSS, version 26 (SPSS, Chicago, IL, USA).

## 3. Results

### 3.1. Patient Population

Based on the inclusion criteria, a total of one hundred and eighty-nine (*n* = 189) patients with AdHF were identified to be potentially included in the study. Fifty-one patients were excluded because of a poor acoustic window or incomplete echocardiographic examination (30), unavailable brachial artery cuff pressure (6), atrial fibrillation (8), and relevant mitral or aortic regurgitation (7). A total of 138 patients were finally included in the study, mostly men (110, 79.7%) with a median age of 58 years (IQR: 50–62). The etiology of AdHF was predominantly non-ischemic (65.9%) and the majority of patients were in NYHA classes II (60.1%) and III (26.8%). The population was divided into two groups according to primary endpoint occurrence. Table 1 shows the clinical characteristics of the study population, including their pharmacological therapy and laboratory results. 

### 3.2. Patients with Composite Endpoints Versus No Composite Endpoints

The median follow-up duration was 636 days (IQR: 323–868). Approximately one quarter of the patients (35, 25.4%) had primary composite endpoints during the follow-ups, and in particular, 6 patients underwent LVAD implantations (4.3%), 18 patients underwent heart transplantation (13.0%) and 16 patients died (11.6%). During the same period, 19 patients (13.8%) needed a new hospitalization for acute HF.

Patients developing the primary endpoint had larger left ventricles, lower LVEF and poorer right ventricular longitudinal function (in terms of tricuspid annular-plane systolic excursion). In addition, the occurrence of events underlined higher left atrial volume, worse chamber longitudinal function (assessed by Peak Atrial Longitudinal Strain, PALS) and higher systolic pulmonary artery pressures (sPAP). Regarding MW parameters, patients with composite events showed lower GWI and GCW and higher GWW (Table 2), providing evidence of poor LV function.

Regarding hospitalization occurrence, patients with a secondary endpoint had lower LVEF and LVGS compared to those without the event (Table 3). Left atrial strain and GCW tended to better in patients with no hospitalization in the follow-up, with a *p*-value at the upper range of statistical significance.

### 3.3. Prognostic Analysis

The results of the univariate and multivariate Cox regression analysis for the composite endpoint are showed in Table 4. Even if some parameters were significantly correlated with the primary endpoint, none of the diastolic function indices, the sPAP, LV GLS and LV MW indices, were associated with major events in the multivariate analysis. Supplementary Table S1 shows the results of the univariate Cox regression analysis for each of the components of the composite endpoint.

As described in Table 5, Cox regression analysis of the secondary endpoint showed that the HR for each increase in GWI by 50 mmHg% was 0.90 (95% CI: 0.78–0.95, *p* = 0.025) and for each increase in GCW by 50 mmHg% was 0.90 (95% CI: 0.82–0.95, *p* = 0.022). LV GLS, LV GWI and LV GCW were significantly associated with the occurrence of the secondary endpoint.

**Figure 1:**
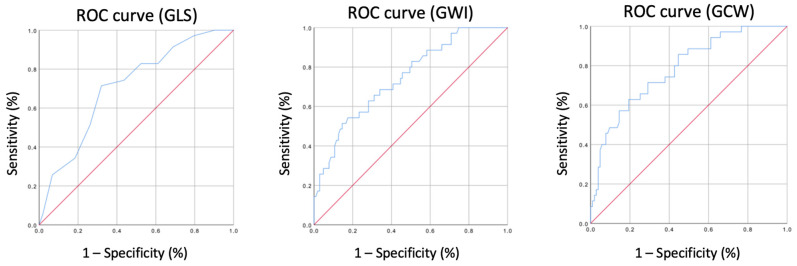
ROC curve of LV global longitudinal strain (GLS), LV global work index (GWI) and LV global constructive work (GCW) for the composite endpoint.

The performance values for the prediction of the primary composite endpoint, tested by the ROC curves, were as follows: LV GLS AUC = 0.709; LV GWI AUC = 0.745; LV GCW AUC = 0.783; LV GWE AUC = 0.569; LV GWW AUC = 0.636 (Figure 1). The performance values for the prediction of the secondary endpoint were as follows: LV GLS AUC = 0.652; LV GWI AUC= 0.632; LV GCW AUC= 0.636; LV GWE AUC= 0.521; LV GWW= 0.540.

The derived optimal cut-off value of LV GWI was 369 mmHg% and for LV GCW was 613 mmHg% for the prediction of the composite endpoint.

### 3.4. Survival Analysis

**Figure 2:**
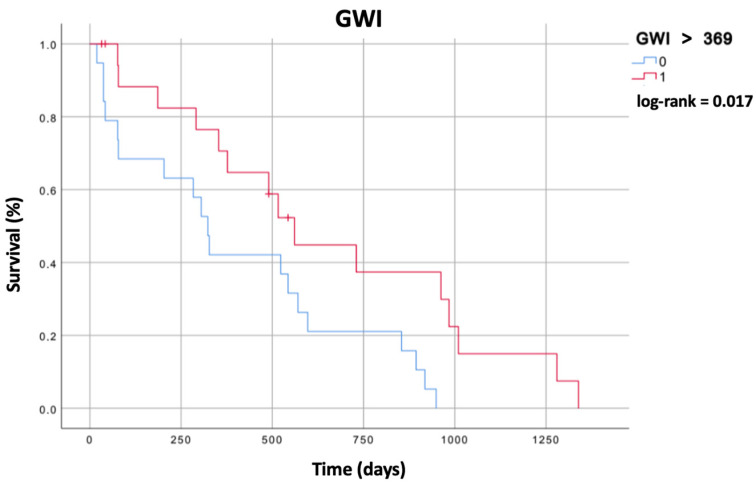
Event-free survival for global work index.

The Kaplan–Meier analysis demonstrated that patients with an LV GWI ≥ 369 mmHg% had a better prognosis in terms of combined endpoint-free survival compared to patients with lower values (log-rank = 0.017) (Figure 2). On the contrary, the Kaplan–Meier analysis did not show better long-term prognoses free of composite endpoints for patients with values of LV GCW ≥ 613 mmHg% (log-rank = 0.337).

## 4. Discussion

In a cohort of HF patients with reduced LV EF referred to our center for AdHF therapies, we found the following: (1) Patients with more severely reduced LV EF, lower PALS and increased pulmonary pressures are associated with the composite event of LVAD implantation, HTX or death. (2) LV EF, LV GLS and PALS are also lower in patients needing hospitalization for acute HF during follow-up. (3) Among the MW indices, GWI and GCW are helpful for the prediction of hospitalization risk; a GWI ≥ 369 mmHg% is the best cut-off for prognostic stratification of event-free survival.

The prevalence of HF is constantly increasing, and the rate of patients reaching advanced stages of the disease is growing accordingly [1,2,3,4], mostly as a consequence of improved drug and electrical therapies. The natural history of the disease is often unpredictable despite the significant amount of research and findings available in the literature [1,6]. Echocardiography remains the cornerstone for managing HF patients both in ambulatory and hospital settings [7,8], being able to provide unique information on cardiac structure and function as well as intracardiac pressures. MW is a relatively novel echocardiographic method created for the quantification of strain (by STE)–pressure loops as a surrogate of pressure–volume loops. Accounting for the LV afterload, which is arterial blood pressure, in the calculation, MW solves the afterload GLS dependence and provides deeper insights into overall heart function and ventricular–arterial coupling [10]. Therefore, in this study, we tested the hypothesis that MW could provide additional information in terms of the prediction of clinically relevant outcomes and adverse events in patients with AdHF. In fact, HF therapies have a strong impact on BP values, with an overall trend of hypotension, and this can interfere with LV performance. To our knowledge, this is the study with the largest population of AdHF patients assessed by MW analysis.

We defined AdHF patients as those referred to our center for advanced HF therapies (i.e., LVAD implantation and heart transplant) and not according to the ESC Guideline definition [1,5]. As such, most of our patients were in a good functional class, different from the required NYHA III/IV class for the definition of AdHF. In our practice, it is customary to evaluate patients’ candidacy for AdHF therapies in a relatively earlier stage of the disease, in order to be able to perform all the cardiac and non-cardiac assessments needed in due time before rapid hemodynamic deterioration occurs. In fact, a previous study from Hertwig F et al. [23] used the same enrolment criteria, also applying comparable endpoints. The authors found a comparable overall rate of events even if in our cohort, and we observed a relatively higher number of patients who died or required HTX, and a lower level of LVAD implantation. The occurrence of unplanned HF hospitalization as a secondary outcome was 13.8% at the latest available follow-up of our population. The relatively low rate of hospitalizations might be explained by the used definition of AdHF, with good functional status of the overall population. In addition, we are used to planning a close follow-up in this setting. It should be mentioned that our treatment strategy for these patients always favors managing them as outpatients rather in hospitals, whenever possible.

Differently from previous data [23], the regression analysis did not show a statistically significant association between MW parameters and the composite outcome. However, the performance of LV GWI and LV GCW in the prediction of the composite outcome was moderate (0.7 < AUC < 0.9). Optimal cut-off values for GWI and GCW were identified from the ROC curves at 369 mmHg% and at 613 mmHg%, respectively, which are both severely reduced compared to the normal reference values. The cut-off of GWI that we identified performed better in our cohort of patients compared to the ones identified by Hedwig F et al., but the cut-off for GCW proposed in that study performed better than ours in risk-stratifying the population for the composite outcome. With regard to the secondary outcome, we hypothesized that unscheduled HF hospitalization could better reflect disease progression in contrast to the single components of the composite outcome. In fact, their occurrence could be influenced by many competing factors which may be unrelated to the actual severity of the disease. Interestingly, the regression analysis showed a significant association between LV GWI, LV GCW and LV GLS with the secondary outcome, with those with lower LV GWI and LV GCW and higher LV GLS being more susceptible to unscheduled HF hospitalization. This result partially gives credit to our hypothesis regarding the more informative data provided by hospitalizations rather than death, HTx and LVAD implantation in terms of disease stage and progression. If these results are confirmed in a larger multicenter study, MW indices might help in the planning of clinical visits or ambulatory diuretics or inotropes administration to prevent hospitalization events.

Our study has some limitations. First, this is a single-center retrospective analysis with a limited sample size. Therefore, our findings cannot be generally applied until they are confirmed in a larger cohort, preferably based on multicenter practices. However, to our knowledge, this is the largest population of AdHF patients assessed by means of MW analysis. Secondly, as already mentioned, we did not use the guideline definition of advanced HF but considered all patients referred for advanced HF therapies as having the condition.

### Clinical Implications

The clinical course of AdHF is characterised by recurrent episodes of congestion or low cardiac output leading to frequent hospitalizations, which reduce the quality of life of this population. Research on new indices able to identify patients at risk of adverse events is needed for the optimization of their management. For example, some outpatients can benefit from the periodical intravenous administration of endo-venous loop diuretics or levosimendan. In addition, the new availability of drugs for worsening HF, i.e., vericiguat, requires a more accurate descriptions of patients’ clinical profiles, and echocardiography should help with this purpose.

## 5. Conclusions

In our pilot study, the use of MW parameters in outpatients referred for AdHF therapies could provide additional information for risk-stratifying patients and enable tailored management in terms of therapeutic optimization and the frequency of follow-up visits. In particular, GWI and GCW seem to be the most useful for this purpose. Our data and future research are paramount to avoid losing the golden hourin research on end-stage strategies such as LVAD implantation and HTx.

## Figures and Tables

**Table 1:** Clinical characteristics of the study population.

	All Patients (*n* = 138)	Composite Endpoint (*n* = 35)	No Composite Endpoint (*n* = 103)	*p*-Value
Age (years)	58 [50–62]	60 [52–64]	57 [50–62]	0.169
Gender				0.307
Male, *n* (%)	110 (80%)	30 (85%)	80 (78%)	
Female, *n* (%)	28 (20%)	5 (15%)	23 (22%)	
Smoking Status				0.674
Non-smoker, *n* (%)	83 (61%)	19 (56%)	64 (62%)	
Active, *n* (%)	19 (13%)	5 (12%)	14 (14%)	
Former, *n* (%)	36 (26%)	11 (32%)	25 (24%)	
Hyperlipidemia, *n* (%)	101 (73%)	27 (76%)	74 (72%)	0.541
Diabetes mellitus, *n* (%)	35 (26%)	8 (23%)	27 (26%)	0.693
Arterial hypertension, *n* (%)	31 (22%)	4 (11%)	27 (26%)	0.081
Obesity (BMI > 30 kg/m^2^), *n* (%)	35 (25%)	8 (23%)	27 (26%)	0.693
CKD (eGFR < 60 mL/min/1.73 m^2^), *n* (%)	25 (18%)	11 (31%)	14 (14%)	**0.018**
Etiology				0.428
Ischemic, *n* (%)	47 (34%)	10 (29%)	37 (36%)	
Non-ischemic, *n* (%)	91 (66%)	25 (71%)	66 (64%)	
NYHA class				**<0.001**
I, *n* (%)	16 (12%)	1 (3%)	15 (15%)	
II, *n* (%)	83 (60%)	15 (43%)	68 (66%)	
III, *n* (%)	37 (27%)	17 (49%)	20 (19%)	
IV, *n* (%)	2 (2%)	2 (6%)	0 (0%)	
ICD at baseline, *n* (%)	122 (88%)	32 (91%)	90 (87%)	0.275
CRT at baseline, *n* (%)	76 (55%)	20 (57%)	56 (54%)	0.650
ARB/ACEi, *n* (%)	63 (46%)	16 (46%)	47 (46%)	0.993
MRA, *n* (%)	126 (91%)	33 (95%)	93 (90%)	0.469
β-blockers, *n* (%)	135 (98%)	35 (100%)	100 (97%)	0.307
ARNI, *n* (%)	61 (44%)	14 (40%)	47 (45%)	0.562
Ivabradine, *n* (%)	18 (13%)	5 (14%)	13 (13%)	0.801
Loop diuretic, *n* (%)	119 (86%)	34 (97%)	85 (82%)	**0.030**
Digoxin, *n* (%)	22 (16%)	7 (20%)	15 (15%)	0.448
SGLT2i, *n* (%)	8 (6%)	1 (3%)	7 (7%)	0.389
Hemoglobin (g/dL)	14 [13–15]	14 [14–15]	14 [13–15]	0.259
Serum creatinine (mg/dL)	1.0 [1.0–1.3]	1.2 [1.0–1.4]	1 [0.8–1.2]	**0.001**
Total bilirubin (mg/dL)	0.5 [0.4–0.8]	0.8 [0.5–1.4]	0.5 [0.4–0.7]	**<0.001**
GOT (UI/L)	20 [17–24]	23 [18–27]	19 [16–23]	**0.009**
GPT (UI/L)	20 [15–26]	20 [15–30]	19 [15–26]	0.493
NT-proBNP (pg/mL)	919 [410–2007]	2419 [1405–6609]	645 [288–1253]	**<0.001**
Serum iron (μg/dL)	81 ± 29	73 ± 25	83 ± 30	0.082
Systolic blood pressure (mmHg)	105 [95–115]	95 [90–110]	105 [100–120]	**0.004**
Diastolic blood pressure (mmHg)	65 [60–75]	60 [60–70]	70 [60–75]	**0.041**

Data are expressed as *n* (%), mean ± SD or median [IQR]. ACEi = angiotensin-converting enzyme (ACE) inhibitor; ARB = angiotensin receptor blocker; ARNI = angiotensin receptor/neprilysin inhibitor; BMI = body mass index; CKD = chronic kidney disease; CRT = cardiac resynchronization therapy; eGFR = estimated glomerular filtration rate; GOT = glutamic–oxaloacetic transaminase; GPT = glutamic–pyruvic transaminase; ICD = implantable cardioverter-defibrillator (ICD); NYHA = New York Heart Association; MRA = mineralocorticoid receptor antagonist; SGLT2i = sodium glucose cotransporter 2 inhibitors.

**Table 2:** Standard and advanced Speckle Tracking Echocardiography-derived echocardiographic indices in the study population and according to composite primary endpoint.

	All Patients (*n* = 138)	Composite Endpoint (*n* = 35)	No Composite Endpoint (*n* = 103)	*p*-Value
LV EDD (mm)	67 ± 10	72 ± 11	66 ± 10	**0.004**
LV EF (%)	30 [23–35]	23 [20–28]	30 [25–37]	**<0.001**
LA volume (mL)	99 [77–127]	121 [94–138]	93 [72–116]	**0.001**
PALS (%)	12.7 [7.9–19.5]	8.8 [6.7–13.1]	15.0 [9.7–21.5]	**0.004**
E/A	0.89 [0.65–1.45]	1.50 [0.74–2.98]	0.82 [0.65–1.37]	**0.018**
E/e’	11 [8–15]	15 [9–22]	11 [7–14]	0.064
sPAP (mmHg)	32 [25–45]	46 [32–55]	30 [25–37]	**0.001**
TAPSE (mm)	18 ± 4	17 ± 4	19 ± 4	**0.043**
LV GLS (%)	−7 [−11–−5]	−5 [−8–−3]	−8 [−11–−5]	**<0.001**
LV GWE (%)	76 ± 11	74 ± 11	77 ± 11	0.202
LV GWI (mmHg%)	598 [353–867]	346 [239–612]	660 [424–908]	**<0.001**
LV GCW (mmHg%)	800 [587–1107]	573 [433–803]	939 [653–1184]	**<0.001**
LV GWW (mmHg%)	196 [138–282]	168 [97–229]	209 [142–287]	**0.016**

Data are expressed as mean ± SD or median [IQR]. EDD = end-diastolic diameter; EF = ejection fraction; LA = left atrial; LV = left ventricular; GLS = global longitudinal strain; GCW = global constructive work; GWE = global work efficiency; GWI = global work index; GWW = global wasted work; PALS = peak atrial longitudinal strain; sPAP = systolic pulmonary artery pressure; TAPSE = tricuspid annular-plane systolic excursion.

**Table 3:** Comparison of standard and advanced STE-derived echocardiographic indices of patients with and without secondary endpoint.

	Secondary Endpoint (*n* = 19)	No Secondary Endpoint (*n* = 119)	*p*-Value
LVEDD (mm)	70 ± 9	67 ± 10	0.167
LVEF (%)	25 [20–30]	30 [23–35]	**0.039**
LA volume (mL)	126 [90–156]	95 [75–121	0.084
PALS (%)	7.2 [5.5–17.0]	13.4 [9.0–19.6]	**0.050**
E/A	1.11 [0.72–1.88]	0.89 [0.63–1.42]	0.839
E/e’	15 [10–19]	11 [7–15]	0.114
sPAP (mmHg)	40 [30–55]	30 [25–43]	0.084
TAPSE (mm)	17 ± 3	18 ± 4	0.194
LV GLS (%)	−6 [−8–−4]	−8 [−11–−5]	**0.033**
LV GWE (%)	76 ± 9	76 ± 11	0.849
LV GWI (mmHg%)	481 [287–651]	609 [362–880]	0.065
LV GCW (mmHg%)	614 [573–970]	814 [592–1182]	**0.050**
LV GWW (mmHg%)	167 [89–293]	198 [139–280]	0.575

Data are expressed as mean ± SD or median [IQR]. EDD = end-diastolic diameter; EF = ejection fraction; LA = left atrial; LV = left ventricular; GLS = global longitudinal strain; GCW = global constructive work; GWE = global work efficiency; GWI = global work index; GWW = global wasted work; PALS = peak atrial longitudinal strain; sPAP = systolic pulmonary artery pressure; TAPSE = tricuspid annular-plane systolic excursion.

**Table 4:** Cox regression analysis for the combined endpoints.

	Univariate		Multivariate	
	HR (CI 95%)	*p*-Value	HR (CI 95%)	*p*-Value
Creatinine	1.93 (0.75–4.96)	0.172		
CKD	4.13 (1.71–9.97)	**0.002**		
Bilirubin	2.06 (1.16–3.66)	**0.013**		
GOT	0.99 (0.98–1.01)	0.868		
NYHA		**0.031**		
I	1.00	/		
II	2.91 (0.36–23.58)	0.316	2.79 (0.92–8.44)	0.069
III	8.50 (0.96–74.96)	0.054	0.89 (0.36–2.17)	0.797
IV	11.13 (0.85–146.59)	0.067		
NTproBNP	1.00 (1.00–1.00)	**<0.001**		0.103
EDD	0.98 (0.95–1.02)	0.375		
LV EF	(0.97–1.06)	0.624	2.95 (0.36–24.51)	0.316
E/A	1.23 (0.87–1.73)	0.244	6.35 (0.64–63.42)	0.115
LA volume	1.00 (0.99–1.02)	0.442	17.44 (1.15–264.16)	0.039
PALS	0.95 (0.86–1.05)	0.293	1.00 (1.00–1.00)	0.166
E/e’	1.00 (0.96–1.03)	0.833		
sPAP	1.02 (0.99–1.05)	0.149		
Systolic BP	1.00 (0.98–1.02)	0.893		
Diastolic BP	0.99 (0.95–1.04)	0.691		
Loop diuretic	0.84 (0.11–6.27)	0.861		
LV GLS	1.03 (0.89–1.19)	0.676		
LV GWE	0.95 (0.90–1.05)	0.422		
LV GWI 50 mmHg%	0.95 (0.90–1.05)	0.326		
LV GCW 50 mmHg%	1.00 (0.90–1.05)	0.599		
LV GWW 50 mmHg%	1.11 (0.90–1.35)	0.316		

BP = blood pressure; CKD = chronic kidney disease; EDD = end-diastolic diameter; EF = ejection fraction; LA = left atrial; LV = left ventricular; GLS = global longitudinal strain; GCW = global constructive work; GWE = global work efficiency; GOT = glutamic–oxaloacetic transaminase; GWI = global work index; GWW = global wasted work; NYHA = New York Heart Association; PALS = peak atrial longitudinal strain; sPAP = systolic pulmonary artery pressure; TAPSE = tricuspid annular-plane systolic excursion.

**Table 5:** Univariate Cox regression analysis for the secondary endpoint.

	Univariate	
	HR (CI 95%)	*p*-Value
LV EF	0.92 (0.85–0.98)	**0.037**
LV GLS	1.19 (1.02–1.40)	**0.028**
LV GWE	0.99 (0.94–1.04)	0.718
LV GWI 50 mmHg%	0.90 (0.78–0.95)	**0.025**
LV GCW 50 mmHg%	0.90 (0.82–0.95)	**0.022**
LV GWW 50 mmHg%	0.95 (0.78–1.16)	0.583

LV = left ventricular; GLS = global longitudinal strain; GCW = global constructive work; GWE = global work efficiency; GWI = global work index; GWW = global wasted work.

## Data Availability

Dataset available on request from the authors.

## Supplementary Materials

The following supporting information can be downloaded at: https://www.mdpi.com/article/10.3390/diagnostics14111196/s1, Table S1: Univariate Cox regression analysis for each of the components of the composite endpoint.
