# Supplementary material for: Myocardial Work Indices Predict Hospitalization in Patients with Advanced Heart Failure
Source: Diagnostics (Basel). 2024 Jun 6;14(11):1196. doi: 10.3390/diagnostics14111196 (PMC11172362; doi:10.3390/diagnostics14111196)
Supplement: Supplementary file 1 [file diagnostics-14-01196-s001.zip › diagnostics-3016600-supplementary.pdf]

Table S1. Univariate Cox regression analysis for each of the components of the composite endpoint

All cause death	Univariate	
	HR (CI 95%)	p-value
LV GLS	1.09 (0.79–1.51)	0.591
LV GWE	0.98 (0.92–1.04)	0.436
LV GWI 50mmHg%	0.90 (0.74–1.11)	0.315
LV GCW 50mmHg%	0.95 (0.82–1.11)	0.350
LV GWW 50mmHg%	0.90 (0.61–1.28)	0.516

LVAD implantation	Univariate	
	HR (CI 95%)	p-value
LV GLS	0.93 (0.65–1.33)	0.696
LV GWE	1.02 (0.95–1.10)	0.563
LV GWI 50mmHg%	1.11 (0.90–1.28)	0.420
LV GCW 50mmHg%	1.16 (0.86–1.57)	0.349
LV GWW 50mmHg%	1.05 (0.58–1.91)	0.874

Heart transplant	Univariate	
	HR (CI 95%)	p-value
LV GLS	0.95 (0.79–1.14)	0.560
LV GWE	0.98 (0.93–1.03)	0.416
LV GWI 50mmHg%	1.00 (0.90–1.11)	0.996
LV GCW 50mmHg%	1.05 (0.90 - 1.16)	0.559
LV GWW 50mmHg%	1.22 (0.90–1.57)	0.213

LV = left ventricular; GLS = global longitudinal strain; GCW = global constructive work; GWE = global work efficiency; GWI = global work index; GWW = global wasted work.
